# Supplementary material for: Formation of a transient amorphous solid in low density aqueous charged sphere suspensions
Source: Sci Rep. 2017 Dec 6;7:17044. doi: 10.1038/s41598-017-17106-6 (PMC5719089; doi:10.1038/s41598-017-17106-6)
Supplement: Supplementary file 1 — supplementary information [file 41598_2017_17106_MOESM1_ESM.pdf]

Formation of a transient amorphous solid in low density aqueous charged sphere suspensions

Ran Niu,[1,*] Sabrina Heidt,[1,2] Ramsia Sreij,[3] Riande I. Dekker,[4] Maximilian Hofmann,[1] and Thomas Palberg[1]

[1] Institute of Physics, Johannes Gutenberg University, D-55099 Mainz, Germany

[2] Graduate School Materials Science in Mainz, Staudinger Weg 9, D-55128 Mainz, Germany

[3] Department of Chemistry Physical and Biophysical Chemistry (PC III), Bielefeld University, D-33615 Bielefeld, Germany

[4] Debye Institute for Nanomaterials Science, Utrecht University, NL-3584 CC Utrecht, The Netherlands

Correspondence to ranniu@uni-mainz.de

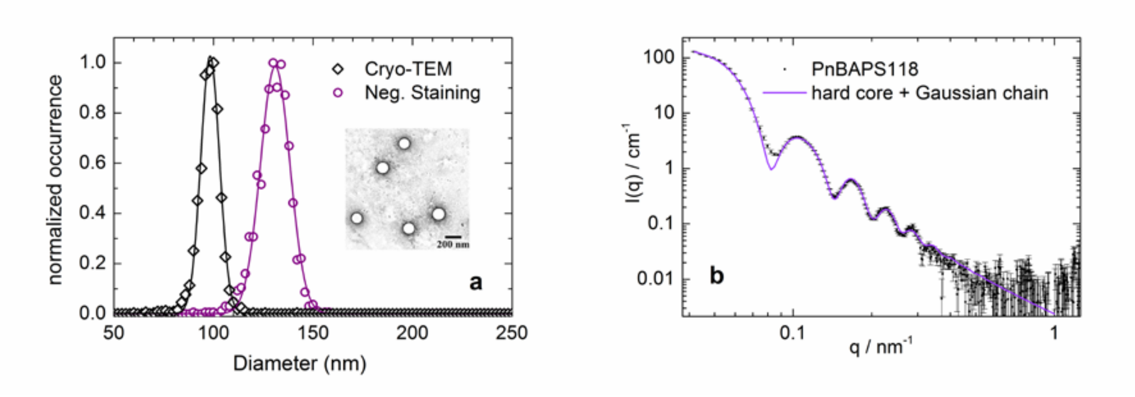

Figure S1. Size and polydispersity determination. a) Results of TEM measurements. For each method, some 1500 particles were imaged, tagged manually to exclude doublets and other artefacts. Sizing was done by standard software. Due to a failure of the absolute calibration, both TEM data sets could only be evaluated for relative widths of the distributions. The solid lines are fittings with Gaussian distribution function and the fitted PI are 0.047 and 0.056, for Cryo-TEM and Negative Staining TEM, respectively. The inset shows an example of a Negative Staining TEM image. b) SAXS scattering curve. The solid line is the fit of the hard core plus Gaussian chains model which returns PI = 0.051, $2a_{\mathrm{SAXS}}$ = 109.1 nm and d = 1 nm.

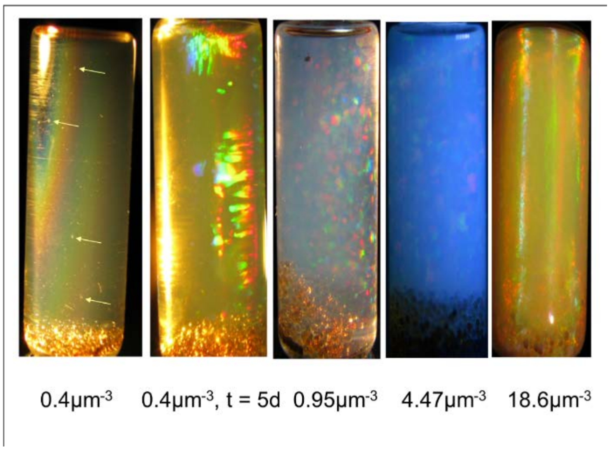

Figure S2. Images of PnBAPS118 samples (illuminated from aside). These were taken at different indicated number densities, *n*, increasing from left to right. Sample height is ca. 35 mm. The leftmost sample is an amorphous solid, as recognized from the pronounced double rainbow and the non-sedimenting ion exchange resin splinters marked by the arrows. After five days, columnar crystals have grown into the amorphous solid after heterogeneous nucleation at the cell wall. At larger number densities homogeneously nucleated crystals are obtained. Note the strong multiple scattering present in the samples at larger *n* ($n > 1\ \mu m^{-3}$) which renders these milky to opaque. (reprinted with permission from Ref. S1. © SISSA Medialab Srl. Reproduced by permission of IOP Publishing. All rights reserved.)

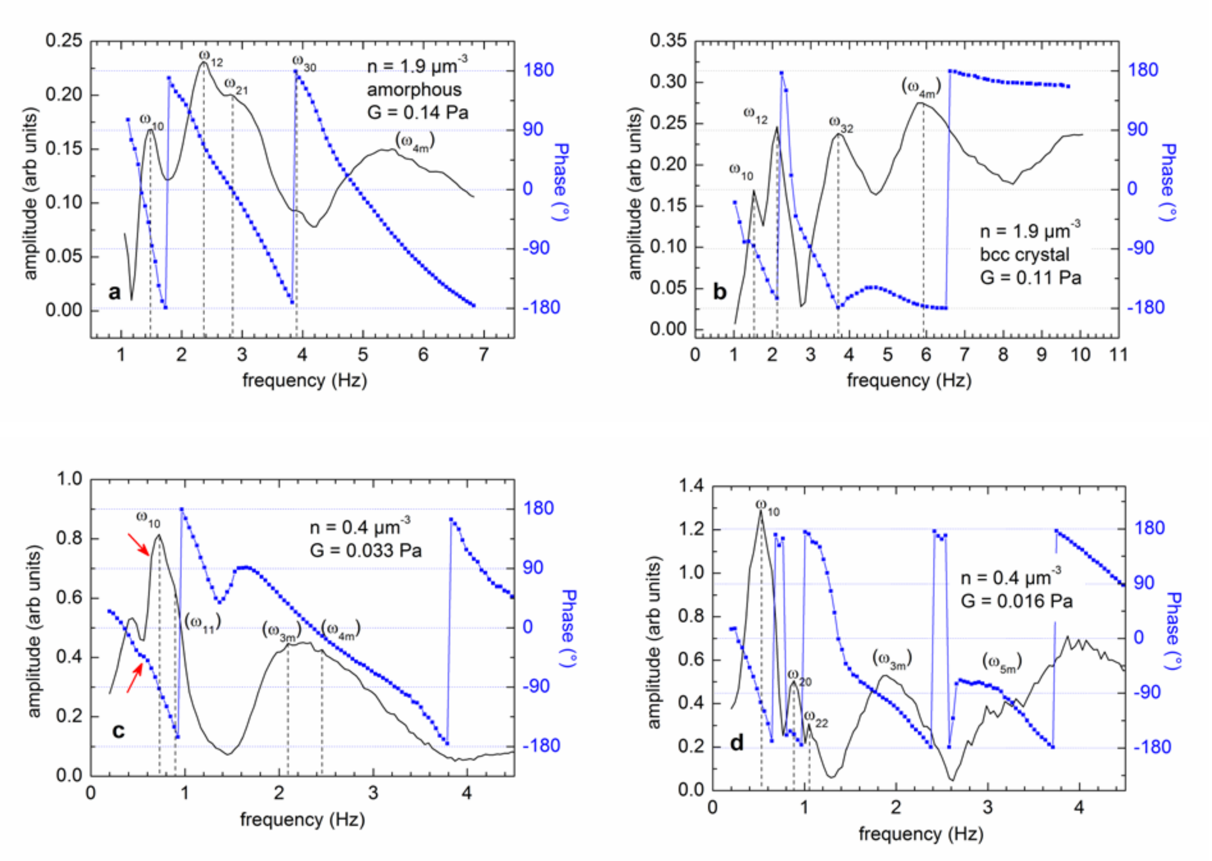

Figure S3. TRS spectra. Data shown for samples at $n = 1.9\ \mu m^{-3}$ (a and b) and $n = 0.4\ \mu m^{-3}$ (c and d) in amorphous (a and c) and crystalline states (b and d). The red arrows in (c) labels a glitch occurring at strong resonant vibration. Immediately afterwards, another frequency sweep was performed and the corresponding spectrum is shown in (d) where the resonance peaks

are sharpened but shifted to lower frequencies as compared with (c). The resonance frequencies are identified with the mode indices indicated. The derived shear moduli are indicated in the figures.

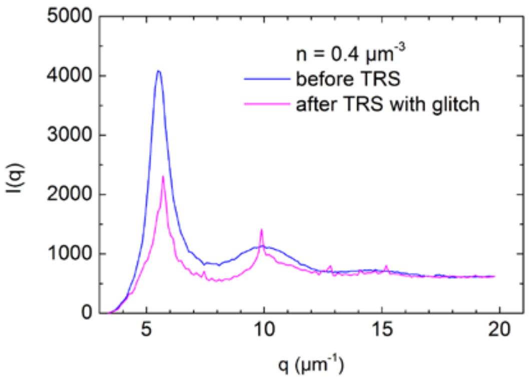

Figure S4. Static light scattering pattern of a sample at n = 0.4 μm^{-3}. Shown are data recorded after leaving the sample undisturbed for several hours and after performing a TRS experiment with a glitch occurring at resonance. The latter data clearly show the onset of crystallization.

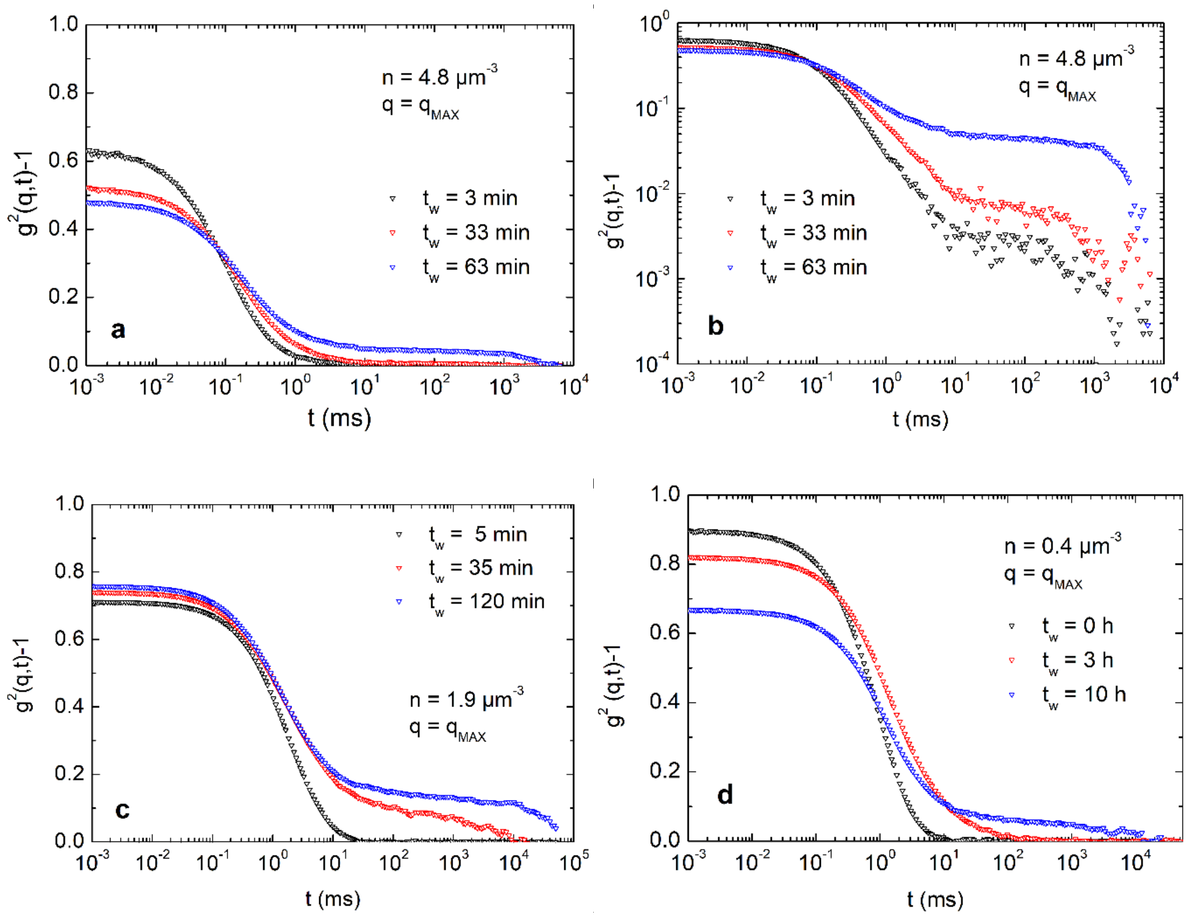

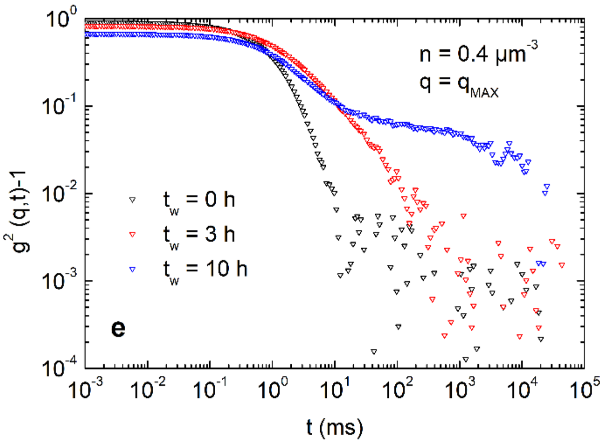

Figure S5. Normalized intensity autocorrelation functions $g^2(q,t)$-1. Data are plotted in both linear-logarithmic fashion (a, c and d) and double-logarithmic fashion (b and e) for vitrifying samples at different number densities of $n = 4.8$ µm^{-3} ($\Phi = 3.8 \times 10^{-3}$), $n = 1.9$ µm^{-3} ($\Phi = 1.3 \times 10^{-3}$) and $n = 0.4$ µm^{-3} ($\Phi = 2.7 \times 10^{-4}$) as indicated. Measurements are shown for different waiting times. All measurements were performed at the respective q_{MAX} of $q_{\mathrm{MAX}} = 11.77$ µm^{-1}, 8.46 µm^{-1} and 5.39 µm^{-1}, respectively.

References

1. Palberg, T., Bartsch, E., Beyer, R., Hofmann, M., Lorenz, N., Marquis, J., Niu, R. & Okubo, T. To make a glass – avoid the crystal. *J. Stat. Phys.* **2016**, 074007; DOI: https://doi.org/10.1088/1742-5468/2016/07/074007 (2016)
